# PPARA Intron Polymorphism Associated with Power Performance in 30-s Anaerobic Wingate Test

**DOI:** 10.1371/journal.pone.0107171

**Published:** 2014-09-08

**Authors:** Miroslav Petr, Petr Št‘astný, Ondřej Pecha, Michal Šteffl, Ondřej Šeda, Eva Kohlíková

**Affiliations:** 1 Department of Physiology and Biochemistry, Faculty of Physical Education and Sport, Charles University in Prague, Prague, Czech Republic; 2 Department of Sport Games, Faculty of Physical Education and Sport, Charles University in Prague, Prague, Czech Republic; 3 Department of Kinanthropology and Humanities, Faculty of Physical Education and Sport, Charles University in Prague, Prague, Czech Republic; 4 Institute of Biology and Medical Genetics, the First Faculty of Medicine, Charles University and the General Teaching Hospital, Prague, Czech Republic; Sanjay Gandhi Medical Institute, India

## Abstract

To date, polymorphisms in several genes have been associated with a strength/power performance including alpha 3 actinin, ciliary neurotrophic factor, vitamin D receptor, or angiotensin I converting enzyme, underlining the importance of genetic component of the multifactorial strength/power-related phenotypes. The single nucleotide variation in peroxisome proliferator-activated receptor alpha gene (*PPARA*) intron 7 G/C (rs4253778; g.46630634G>C) has been repeatedly found to play a significant role in response to different types of physical activity. We investigated the effect of *PPARA* intron 7 G/C polymorphism specifically on anaerobic power output in a group of 77 elite male Czech ice hockey players (18–36 y). We determined the relative peak power per body weight (P_max_.kg^−1^) and relative peak power per fat free mass (W.kg^−1^_FFM_) during the 30-second Wingate Test (WT30) on bicycle ergometer (Monark 894E Peak bike, MONARK, Sweden). All WT30s were performed during the hockey season. Overall genotype frequencies were 50.6% GG homozygotes, 40.3% CG heterozygotes, and 9.1% CC homozygotes. We found statistically significant differences in P_max_.kg^−1^ and marginally significant differences in P_max_.kg^−1^_FFM_ values in WT30 between carriers and non-carriers for C allele (14.6±0.2 vs. 13.9±0.3 W.kg^−1^ and 15.8±0.2 vs. 15.2±0.3 W.kg^−1^_FFM_, P = 0.036 and 0.12, respectively). Furthermore, P_max_.kg^−1^_FFM_ strongly positively correlated with the body weight only in individuals with GG genotypes (R = 0.55; p<0.001). Our results indicate that *PPARA* 7C carriers exhibited higher speed strength measures in WT30. We hypothesize that C allele carriers within the cohort of trained individuals may possess a metabolic advantage towards anaerobic metabolism.

## Introduction

The study of genomic component of traits comprising athletic performance is at the center of the highly dynamic field of sports genomics. To date, polymorphisms in several genes have been associated with a strength/power performance including actinin, alpha 3 (*ACTN3)*, ciliary neurotrophic factor (*CNTF)*, vitamin D receptor (*VDR)*, or info angiotensin I converting enzyme (*ACE)* [1]–[4]. The single nucleotide variation in peroxisome proliferator-activated receptor alpha gene (*PPARA*) intron 7 G/C (rs4253778; g.46630634G>C) has been repeatedly found to play a significant role in response to physical activity and other relevant gene-environment interactions [5]. Peroxisomes are organelles within the cell that play an important role in metabolism of fatty acids. They provide several essential metabolic functions such as shortening of very-long-chain fatty acids, later to be degraded in the mitochondria, and also help the cells to get rid of toxic peroxides. Peroxisomes proliferate and decrease in response to dietary lipids, hormones, hypolipidemic drugs, herbicides, and leukotriene antagonists, that bind to nuclear regulatory proteins called peroxisome proliferator-activated receptors (PPARs) proteins, which belong to the steroid hormone receptor superfamily. These receptors combine with the retinoid X receptors to form heterodimers that regulate genes involved in lipid and glucose metabolism, adipocyte differentiation, fatty acid transport, carcinogenesis, and inflammation [6]–[9]. PPARs exist in three different forms as PPAR-alpha (PPARα), PPAR-beta/delta (PPARβ/δ), and PPAR-gamma (PPARγ), which are encoded by similarly named genes – *PPARA*, *PPARD*, and *PPARG* [10]. PPARs act as ligand-activated transcription factors similar to other nuclear hormone receptors. Three variants of PPAR are distributed among various tissues. Unsaturated fatty acids bind to PPARα which is highly expressed in the heart, liver, kidney, and skeletal muscles [10], activating genes involved in fatty acid metabolism. During long-term fasting, the free fatty acids that are mobilized from adipose tissue bind to PPARα, enhancing hepatic fatty acid oxidation and production of ketone bodies, preventing hypoglycemia [11]. PPARα plays a role in the inflammatory response during atherosclerosis [12], [13].

Several studies have associated *PPARA* gene variants with physical activity. For instance, it was associated with left ventricular growth in response to a ten-week exercise program with the largest increase found in 7C homozygotes [14]. Another study focusing on athletes in 13 different sport disciplines showed a linear trend of 7C allele with increasing anaerobic component of physical performance [15]. The analysis of muscle fiber composition in 40 young men revealed higher percentage of slow-twitch fibers in 7G homozygotes compared to 7C homozygotes [15], although a statistical significance could not be reached due to a low frequency of 7C/7C genotypes (n = 4). In another study *PPARA* intron 7 G/C variant along with *ACE* I/D variant were found to be the strongest predictors for muscle fiber type determination [16]. Group of Ahmetov et al. [17] included 7G allele of *PPARA* gene among ten “endurance alleles” whose number positively correlated with the proportion of slow-twitch muscle fibers, and with maximal oxygen consumption. According to Eynon et al. [18] endurance athletes showed a trend of a higher yet not significant proportion of the 7G/7G genotype compared with sprinters. Study of Lithuanian athletes had shown that male athletes with allele 7C had significantly higher muscle mass and better results in explosive strength of lower extremities than 7G/7G homozygotes [19]. On the other hand, 7C/7C genotype was more frequent among athletes in endurance and team sports than in speed/power and mix sports.

The objective of this study is to determine if previous findings of higher frequency of *PPARA* 7C allele in power/strength-oriented athletes would be supported by a positive trend to superior speed and power performance in a group of elite ice hockey players.

## Materials and Methods

### Subjects

All subjects (n = 77) were young, aged 18–36 y old, healthy, physically active Caucasian males – players of I. and II. National Czech ice hockey league. The detailed subject characteristics are shown in Table S1. The players were tested in the middle of the ice hockey season during the one-week competition break. None of our subjects followed low carbohydrate or energy restrictive diet before the tests. The Wingate test (WT30, described in detail below) results are referring to condition in season. Written informed consent was obtained from all subjects under protocols approved by the Institutional Ethics Committee of the Charles University of Faculty of Physical Education and Sport.

### Anthropometric Measurement

Body weight (in kilograms) and height (in centimeters) were assessed prior to Wingate test to calculate an individual constant braking resistance. To estimate body fat percentage (BF) and fat free mass (FFM), the skinfold thickness was measured at seven sites (chest, midaxillary, triceps, subscapular, abdominal, suprailiac, front midthigh) by one trained technician using a Harpenden Skinfold Caliper (Baty International, Burgess Hill, UK) and formula by Jackson and Pollock was used [20].

### Wingate Test

WT30 was used to diagnose an anaerobic power output of the tested group. Power output is calculated as a mean each 5 s. The best 5 s is called peak power, which is related to maximum and explosive strength abilities of individual [21]. The values can either be expressed in absolute values of peak power (P_max_) expressed in Watt (W), relative peak power per body weight (P_max_.kg^−1^) expressed in Watt per body weight (W.kg^−1^) or relative peak power per FFM expressed in Watt per FFM (W.kg^−1^_FFM_). P_max_ is regarded to mirror the alactate anaerobic processes, strength-speed abilities [22] and muscle rheological properties [23] of the tested subject. The WT30 test is a standard laboratory test for ice-hockey players and it is related to their skating performance [21], [24]. The WT30 test was conducted on a calibrated friction loaded cycle ergometer (Monark 894E Peak bike, MONARK, Sweden) interfaced with a microcomputer. The cycle was equipped with toe-clips to prevent the subject’s feet from slipping. The test consisted of a 30 s maximal sprint against a constant braking resistance dependent on the subjects’ body mass (0.091 kg.kg^−1^ body mass) according to the optimization tables of Bar-Or [22]. The test began from a rolling start, at maximal individual repetition against minimal resistance. When the maximum pedal rate was achieved, a countdown of “3-2-1-go!” was given before dropping the weight basket with a load. Prior to the test participants were instructed to pedal as fast as they could for 30 s.

### DNA and Genotyping

Saliva samples were collected from individuals using FTA-cards (Whatman, USA). Samples (3 mm punch) were lysed and DNA was stabilized with DNA Extract All Reagents Kit (Applied Biosystems, USA) according to the manufactureŕs protocol. Selected loci were amplified in thermal cycler (Eppendorf, Germany), using TaqMan SNP Genotyping Assays (Assaýs IDs: C__2985251_10) and TaqMan Genotyping Master Mix (Applied Biosystems), in 20-µl reactions, each containing 10 µl of Master Mix, 1 µl of genotyping assay, 5 µl of DNase-free water and 4 µl of sample lysate. Cycling conditions were as follows: DNA polymerase activation at 95°C for 20 s, followed by 40 cycles of denaturation at 95°C for 3 s and Annealing/Extending step at 60°C for 20 s. PCR products were then subjected to Endpoint-genotyping analysis (program: post-genotyping, analysis mode: melting curves, detection format: dual color hydrolysis probe, color compensation: universal CC FAM(510) – VIC(580)) at LC 480 Light Cycler (Roche), in order to measure the relative amount of allele-specific fluorescence (FAM or VIC), which leads directly to the determination of individual genotypes.

### Statistics

All the phenotype and genotype data are presented in the Supporting information file in form of Table S1. All variables were initially tested for normality using the Kolmogorov-Smirnov test. Since all the variables were normally distributed, the data are expressed as a mean ± SEM (Standard Error of the Mean). One-way analysis of variance (ANOVA) and independent samples t-test were used to evaluate differences between genotype groups. Pearson product-moment correlations and linear regression analyses specific for distinct genotype groups were performed to assess the relationships among continuous variables. Statistical significance was accepted at P<0.05 and p-values<0.1 were considered as marginally significant. All analyses were conducted using SPSS version 17.0 (SPSS, Inc., Chicago, IL).

## Results and Discussion

**Figure 1:**
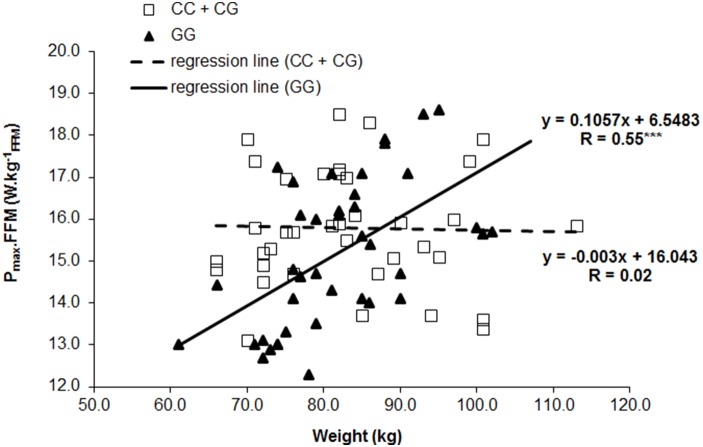
Effect of PPARA intron 7 G/C polymorphism on the relationship between body weight and P_max_.FFM. ***p<0.001; R = Pearson product – moment correlation.

As evident from the Table 1 listing the genotype and allelic frequencies of all 77 ice hockey players, the minor C allele was found at a frequency over 29%. Such frequency is substantially higher than those observed e.g. in reference Caucasian HapMap populations (19.6%), European healthy controls (Poles–21.3%, British–18.1%, Germans–17.9%) [14], [25] and much higher than C allele frequencies detected in endurance-oriented cohorts [25], [26]. The assumption of the Hardy-Weinberg equilibrium was tested using Pearson *χ* ^2^ test. It was found that this test fails to reject the null hypothesis that the population is in Hardy-Weinberg equilibrium (*χ* ^2^ = 0.055; df = 1; p = 0.97). There were no significant differences observed in age, anthropometric characteristics, or activity level across different *PPARA* genotypes (Table 2). We found statistically significant difference of P_max_.kg^−1^ and marginally significant difference of P_max_.kg^−1^_FFM_ in WT30 between carriers and non-carriers for C allele (14.6±0.2 vs. 13.9±0.3 W.kg^−1^ and 15.8±0.2 vs. 15.2±0.3 W.kg^−1^_FFM_, P = 0.036 and 0.12, respectively). The comparison among individual genotypes revealed no significant differences (P = 0.095 and 0.21 for P_max_.kg^−1^ and P_max_.FFM, respectively) possibly reflecting relatively low number of CC homozygous in our group of athletes (N = 7). Furthermore, P_max_.kg^−1^_FFM_ positive correlation with the body weight was significantly stronger in individuals with GG genotypes in comparison with carriers of the C allele (R = 0.55 [GG] vs. 0.02 [CG+CC], respectively; z-score = 2.538; p = 0.0055) as shown in Figure 1.

**Table 1:** PPARA 7 G/C genotype and allelic frequencies in 77 elite hockey players.

	GG	CG	CC	allele G	allele C
Frequencies (%)	0.51	0.40	0.09	0.71	0.29

**Table 2:** Basic descriptive statistics.

Variable	GG	CG	CC	CG + CC	p-value CC + CG vs. GG
N	39	31	7	38	
age	25.6±0.7	24.3±0.7	26.1±1.6	24.7±0.6	0.31
height (cm)	181.8±0.8	180.9±1.2	181.4±3.5	181.0±1.2	0.59
weight (kg)	81.9±1.5	82.2±2.0	86.5±4.9	83.0±1.8	0.63
FFM (kg)	75.0±1.2	76.2±1.7	76.9±3.9	76.3±1.5	0.49
P_max_.kg^−1^(W.kg^−1^)	13.9±0.3	14.7±0.2	14.3±0.6	14.6±0.2	0.036*
P_max_.FFM(W.kg^−1^_FFM_)	15.2±0.3	15.9±0.2	15.3±0.6	15.8±0.2	0.12

Data are expressed as mean ± SEM (Standard Error of the Mean); p-values are based on independent samples t-tests; *p<0.05.

The present report is the first to demonstrate a higher relative peak power per body weight (P_max_.kg^−1^), and relative peak power per FFM (P_max_.kg^−1^_FFM_) in WT30 in *PPARA* 7C allele carriers. Our results corroborate and extend the previous reports showing that 7C is a hot candidate of speed/power-related allele. Ice hockey is characterized by high intensity intermittent skating, rapid changes in velocity and duration, and frequent body contact. The intensity and duration of a particular shift determines the extent of the contribution from aerobic and anaerobic energy systems. Glycogen depletion studies show a preferential utilization of glycogen from the slow twitch fibers but also significant depletion from the fast twitch fibers. Although elite hockey players display a muscle fiber composition similar to untrained individuals [27], those with predominance of fast twitch fibers could have advantage in training of muscular strength and skating speed.

Better scores in WT30 in C allele carriers and positive peak power-increasing effect with body weight in GG genotypes do not have a clear explanation. Activation of PPARA favors lipid sparing [28], improves insulin sensitivity and affects some important cell functions [29]. Although 7 G/C polymorphism is located in noncoding region, it has been associated with a functional variant in a promoter or enhancer element of the *PPARA* gene that results in reduced *PPARA* gene expression [14]. It was shown that C allele carriers are possibly less responsive to β-adrenergic stimulation compared with GG homozygotes [30]. In fact, 7 C/C genotype and C allele containing haplotype significantly increased the risk for diabetes [31], [32]. The consequences of these changes may have an important role in a muscle metabolism, such as inhibited glycogenolysis and partly limited glucose transport into a skeletal muscle. On the other hand, an increased expression of *PPARA* gene and the intermittent increase in fatty acid delivery to muscle during exercise training may both be important factors in enhancing muscle fatty acid oxidative capacity [33]. These processes are likely to be more active in 7G carriers. Under the circumstances it is possible that during intense exercise 7C carriers are more prone to rely on anaerobic processes what may be an adaptive stimulus leading to muscle fiber transformation. A muscle fiber composition have not been monitored in our study, but at least two studies associated *PPARA* 7 G/C genotypes with a muscle fiber specific composition in athletes [15], [16]. Whilst initial composition is likely to be strongly influenced by genetic factors, training has significant effects on fiber shifts and related muscle metabolism changes. We propose an important role of *PPARA* 7 G/C polymorphism in these processes.

We are aware of several limitations of our study including relatively smaller sample size and potential effects of other environmental (e.g. dietary) or genetic factors. Validation in other cohorts and further studies are necessary to address the detailed role of the *PPARA* intron polymorphism within the complex phenotype of strength/power performance. All subjects included in our study were male elite ice hockey players, so whether the results are applicable to female population engaged in the same or similar sport discipline, is uncertain. Also, our findings most likely cannot be generalized to normal male population. Recent study in nonathletic population did not confirm any difference in strength parameters during static or dynamic conditions depending on 7 G/C polymorphism [34].

Our results regarding *PPARA* intron 7 G/C polymorphic site association with anaerobic power output may contribute to better understanding how genetics affects individual physiological variability resulting in different expression of muscle work. The C allele carriers might favor power/strength-oriented sport performance. Once the genetic variants related to athletes’ performance are understood, it may be possible to develop genetic tests that may be used to identify sport talent. Furthermore, intervention genetic studies in the future may possibly uncover what training methods and approaches are potentially more beneficial for different genotypes.

## Supporting Information

Table S1 **Descriptive data of the 77 ice-hockey players.** LBM - lean body mass; Pmax/kg - relative peak power per body weight.
(PDF)
