# Correction: PPARA Intron Polymorphism Associated with Power Performance in 30-s Anaerobic Wingate Test

**DOI:** 10.1371/journal.pone.0134424

**Published:** 2015-07-28

**Authors:** Miroslav Petr, Petr Stastny, Ondřej Pecha, Michal Šteffl, Ondřej Šeda, Eva Kohlíková

The second author’s name is spelled incorrectly. The correct name is: Petr Stastny. The correct citation is: Petr M, Stastny P, Pecha O, Šteffl M, Šeda O, Kohlíková E (2014) PPARA Intron Polymorphism Associated with Power Performance in 30-s Anaerobic Wingate Test. PLoS ONE 9(9): e107171. doi:10.1371/journal.pone.0107171
