# Supplementary material for: PPARA Intron Polymorphism Associated with Power Performance in 30-s Anaerobic Wingate Test
Source: PLoS One. 2014 Sep 8;9(9):e107171. doi: 10.1371/journal.pone.0107171 (PMC4157815; doi:10.1371/journal.pone.0107171)
Supplement: Table S1 — Descriptive data of the 77 ice-hockey players. LBM - lean body mass; Pmax/kg - relative peak power per body weight. (PDF) [file pone.0107171.s001.pdf]

| subject ID | age | height | weight | body fat (kg) | body fat (%) | LBM (kg) | Pmax/kg | Pmax/ATH | *PPARA (rs4253778)* |
|---|---|---|---|---|---|---|---|---|---|
| 1 | 27 | 187 | 102,0 | 9,0 | 8,8 | 93,0 | 14,2 | 15,7 | G/G |
| 2 | 24 | 186 | 76,0 | 4,0 | 5,3 | 72,0 | 13,9 | 14,7 | C/G |
| 3 | 24 | 170 | 66,0 | 4,0 | 6,1 | 62,0 | 14,0 | 15,0 | C/G |
| 4 | 27 | 173 | 72,0 | 6,0 | 8,3 | 66,0 | 12,0 | 13,1 | G/G |
| 5 | 24 | 181 | 81,0 | 6,0 | 7,4 | 75,0 | 13,2 | 14,3 | G/G |
| 6 | 23 | 168 | 61,0 | 4,0 | 6,6 | 57,0 | 12,1 | 13,0 | G/G |
| 7 | 23 | 183 | 84,0 | 4,0 | 4,8 | 80,0 | 15,5 | 16,3 | G/G |
| 8 | 26 | 172 | 75,0 | 6,4 | 8,5 | 68,6 | 14,4 | 15,7 | C/G |
| 9 | 23 | 183 | 82,0 | 7,2 | 8,8 | 74,8 | 14,6 | 16,2 | G/G |
| 10 | 24 | 175 | 71,0 | 5,0 | 7,0 | 66,0 | 14,7 | 15,8 | C/G |
| 11 | 24 | 180 | 85,0 | 6,0 | 7,1 | 79,0 | 12,8 | 13,7 | C/G |
| 12 | 25 | 170 | 72,0 | 5,0 | 6,9 | 67,0 | 12,7 | 14,5 | C/G |
| 13 | 24 | 181 | 76,0 | 5,0 | 6,6 | 71,0 | 13,2 | 14,1 | G/G |
| 14 | 32 | 184 | 94,0 | 8,0 | 8,5 | 86,0 | 12,5 | 13,7 | C/G |
| 15 | 26 | 183 | 88,0 | 10,0 | 11,4 | 78,0 | 15,9 | 17,9 | G/G |
| 16 | 28 | 190 | 88,0 | 6,0 | 6,8 | 82,0 | 16,6 | 17,8 | G/G |
| 17 | 25 | 182 | 81,0 | 10,0 | 12,3 | 71,0 | 15,0 | 17,1 | G/G |
| 18 | 26 | 187 | 95,0 | 10,0 | 10,5 | 85,0 | 16,7 | 18,6 | G/G |
| 19 | 26 | 187 | 82,0 | 4,0 | 4,9 | 78,0 | 16,3 | 17,2 | C/G |
| 20 | 24 | 176 | 79,0 | 5,0 | 6,3 | 74,0 | 15,2 | 16,0 | G/G |
| 21 | 22 | 185 | 85,0 | 4,0 | 4,7 | 81,0 | 16,2 | 17,1 | G/G |
| 22 | 24 | 179 | 82,0 | 5,0 | 6,1 | 77,0 | 16,1 | 17,1 | C/G |
| 23 | 28 | 175 | 70,0 | 7,0 | 10,0 | 63,0 | 16,2 | 17,9 | C/G |
| 24 | 25 | 188 | 99,0 | 7,0 | 7,1 | 92,0 | 16,2 | 17,4 | C/G |
| 25 | 29 | 180 | 86,0 | 6,5 | 7,6 | 79,5 | 16,9 | 18,3 | C/C |
| 26 | 29 | 191 | 100,0 | 7,0 | 7,0 | 93,0 | 14,7 | 15,8 | G/G |
| 27 | 25 | 178 | 82,0 | 4,0 | 4,9 | 78,0 | 17,1 | 18,5 | C/G |
| 28 | 20 | 192 | 100,8 | 10,8 | 10,7 | 90,0 | 16,1 | 17,9 | C/G |
| 29 | 24 | 180 | 80,0 | 6,0 | 7,5 | 74,0 | 15,9 | 17,1 | C/G |
| 30 | 26 | 192 | 93,0 | 10,0 | 10,8 | 83,0 | 16,5 | 18,5 | G/G |
| 31 | 21 | 180 | 72,0 | 4,0 | 5,6 | 68,0 | 12,0 | 12,7 | G/G |
| 32 | 26 | 189 | 85,0 | 5,0 | 5,9 | 80,0 | 13,3 | 14,1 | G/G |
| 33 | 25 | 181 | 79,0 | 4,5 | 5,7 | 74,5 | 11,9 | 14,7 | G/G |
| 34 | 21 | 175 | 70,0 | 2,0 | 2,9 | 68,0 | 12,7 | 13,1 | C/G |
| 35 | 24 | 180 | 73,0 | 4,1 | 5,6 | 68,9 | 12,1 | 12,9 | G/G |
| 36 | 21 | 175 | 71,0 | 3,5 | 4,9 | 67,5 | 12,4 | 13,0 | G/G |
| 37 | 20 | 193 | 78,0 | 3,0 | 3,8 | 75,0 | 11,8 | 12,3 | G/G |
| 38 | 22 | 183 | 84,0 | 8,0 | 9,5 | 76,0 | 14,7 | 16,1 | C/G |
| 39 | 23 | 179 | 72,0 | 5,0 | 6,9 | 67,0 | 13,0 | 15,2 | C/G |
| 40 | 32 | 182 | 79,0 | 3,0 | 3,8 | 76,0 | 13,0 | 13,5 | G/G |
| 41 | 33 | 182 | 95,0 | 10,0 | 10,5 | 85,0 | 14,1 | 15,1 | C/G |
| 42 | 28 | 184 | 90,0 | 10,0 | 11,1 | 80,0 | 12,5 | 14,1 | G/G |
| 43 | 24 | 176 | 90,0 | 14,0 | 15,6 | 76,0 | 12,4 | 14,7 | G/G |
| 44 | 22 | 176 | 66,0 | 4,0 | 6,1 | 62,0 | 13,4 | 14,8 | C/G |
| 45 | 27 | 171 | 83,0 | 4,0 | 4,8 | 79,0 | 14,8 | 15,5 | C/G |
| 46 | 26 | 180 | 77,0 | 7,0 | 9,1 | 70,0 | 14,9 | 16,1 | G/G |
| 47 | 24 | 185 | 82,0 | 7,0 | 8,5 | 75,0 | 14,5 | 15,9 | C/G |
| 48 | 23 | 183 | 82,0 | 4,0 | 4,9 | 78,0 | 15,5 | 16,1 | G/G |
| 49 | 22 | 181 | 72,0 | 5,0 | 6,9 | 67,0 | 13,9 | 14,9 | C/C |
| 50 | 24 | 179 | 84,0 | 10,6 | 12,6 | 73,4 | 12,8 | 16,6 | G/G |
| 51 | 30 | 181 | 76,0 | 9,0 | 11,8 | 67,0 | 13,1 | 14,8 | G/G |
| 52 | 25 | 186 | 97,0 | 6,0 | 6,2 | 91,0 | 15,5 | 16,0 | C/C |
| 53 | 24 | 177 | 80,0 | 5,0 | 6,3 | 75,0 | 15,9 | 17,1 | C/G |
| 54 | 36 | 179 | 91,0 | 21,0 | 23,1 | 70,0 | 13,2 | 17,1 | G/G |
| 55 | 28 | 170 | 73,0 | 3,0 | 4,1 | 70,0 | 14,7 | 15,3 | C/C |
| 56 | 29 | 186 | 86,0 | 6,0 | 7,0 | 80,0 | 13,1 | 14,0 | G/G |
| 57 | 27 | 177 | 75,0 | 4,0 | 5,3 | 71,0 | 12,6 | 13,3 | G/G |
| 58 | 27 | 176 | 76,0 | 8,0 | 10,5 | 68,0 | 13,9 | 15,7 | C/C |
| 59 | 20 | 183 | 77,0 | 4,2 | 5,5 | 72,8 | 13,8 | 14,6 | G/G |
| 60 | 21 | 183 | 93,0 | 7,7 | 8,3 | 85,3 | 14,1 | 15,3 | C/G |
| 61 | 20 | 183 | 89,0 | 6,0 | 6,7 | 83,0 | 14,1 | 15,1 | C/G |
| 62 | 20 | 180 | 75,0 | 4,0 | 5,3 | 71,0 | 16,1 | 17,0 | C/G |
| 63 | 19 | 179 | 81,0 | 6,7 | 8,3 | 74,3 | 14,5 | 15,9 | C/G |
| 64 | 19 | 179 | 74,0 | 4,1 | 5,6 | 69,9 | 16,3 | 17,2 | G/G |
| 65 | 23 | 175 | 71,0 | 3,0 | 4,2 | 68,0 | 16,7 | 17,4 | C/G |
| 66 | 32 | 178 | 100,8 | 28,8 | 28,6 | 72,0 | 12,4 | 13,4 | C/C |
| 67 | 34 | 180 | 86,1 | 9,1 | 10,6 | 77,0 | 13,8 | 15,4 | G/G |
| 68 | 34 | 198 | 113,0 | 11,0 | 9,8 | 102,0 | 14,3 | 15,8 | C/G |
| 69 | 28 | 183 | 90,0 | 9,6 | 10,7 | 80,4 | 14,2 | 15,9 | C/G |
| 70 | 30 | 181 | 76,0 | 6,0 | 7,9 | 70,0 | 15,6 | 16,9 | G/G |
| 71 | 20 | 181 | 85,0 | 5,0 | 5,9 | 80,0 | 14,7 | 15,6 | G/G |
| 72 | 22 | 191 | 83,0 | 5,7 | 6,9 | 77,3 | 15,8 | 17,0 | C/G |
| 73 | 20 | 192 | 87,0 | 7,0 | 8,0 | 80,0 | 13,5 | 14,7 | C/G |
| 74 | 20 | 199 | 100,8 | 10,4 | 10,3 | 90,4 | 13,1 | 13,6 | C/C |
| 75 | 20 | 177 | 66,0 | 2,0 | 3,1 | 64,0 | 14,0 | 14,4 | G/G |
| 76 | 35 | 183 | 100,8 | 18,8 | 18,7 | 82,0 | 14,4 | 15,7 | G/G |
| 77 | 27 | 178 | 74,0 | 5,0 | 6,8 | 69,0 | 12,1 | 13,0 | G/G |

Supplementary Table S1. Descriptive data of the 77 ice-hockey players.
LBM - lean body mass; Pmax/kg - relative peak power per body weight.
